# CropPainter: an effective and precise tool for trait-to-image crop visualization based on generative adversarial networks

**DOI:** 10.1186/s13007-022-00970-3

**Published:** 2022-12-15

**Authors:** Lingfeng Duan, Zhihao Wang, Hongfei Chen, Jinyang Fu, Hanzhi Wei, Zedong Geng, Wanneng Yang

**Affiliations:** National Key Laboratory of Crop Genetic Improvement, Key Laboratory of Agricultural Equipment for the Middle and Lower Reaches of the Yangtze River, Ministry of Agriculture, and College of Engineering, Hubei Hongshan Laboratory, National Center of Plant Gene Research, Huazhong Agricultural University, Wuhan, 430070 People’s Republic of China

**Keywords:** Crop visualization, Virtual plant, Crop phenotypic traits, Deep learning, Generative adversarial network

## Abstract

**Background:**

Virtual plants can simulate the plant growth and development process through computer modeling, which assists in revealing plant growth and development patterns. Virtual plant visualization technology is a core part of virtual plant research. The major limitation of the existing plant growth visualization models is that the produced virtual plants are not realistic and cannot clearly reflect plant color, morphology and texture information.

**Results:**

This study proposed a novel trait-to-image crop visualization tool named CropPainter, which introduces a generative adversarial network to generate virtual crop images corresponding to the given phenotypic information. CropPainter was first tested for virtual rice panicle generation as an example of virtual crop generation at the organ level. Subsequently, CropPainter was extended for visualizing crop plants (at the plant level), including rice, maize and cotton plants. The tests showed that the virtual crops produced by CropPainter are very realistic and highly consistent with the input phenotypic traits. The codes, datasets and CropPainter visualization software are available online.

**Conclusion:**

In conclusion, our method provides a completely novel idea for crop visualization and may serve as a tool for virtual crops, which can assist in plant growth and development research.

**Supplementary Information:**

The online version contains supplementary material available at 10.1186/s13007-022-00970-3.

## Background

With the rapid development of computer technology, the application of computer technology in agriculture has gradually increased [1–3]. Benefiting from the development of digital agriculture, the concept of virtual plants [4] was proposed and gained the attention of a large number of researchers. The virtual plant is a multidisciplinary technology that combines multiple types of knowledge, including computer graphics, botany, geography, agriculture, and virtual reality [5]. Virtual plants simulate plant growth and development processes through computer modeling so that they can be analyzed and predicted on the computer [6, 7]. As one of the important research areas in digital agricultural technology, virtual plants play an important role in plant cultivation, strain improvement, plant growth and yield prediction and thus are of great significance in revealing plant growth and development patterns [8]. Virtual plant visualization is an important part of virtual plants [9, 10]. The main purpose of virtual plant visualization is to simulate plant morphology and growth through computer modeling [11].

The study of virtual crop visualization was proposed early by researchers. The L-system [12], first proposed by Lindenmayer in 1968, was essentially a theory that continuously updated the strings representing plant growth and added geometric interpretations to the strings to generate fractal graphs of plants. In later studies, the L-system was continuously improved and extended [13–18]. Allen et al. proposed the L-peach model for modeling structural and physiological changes during tree growth [19]. Leitner et al. modeled plant root growth based on the L-system by defining growth rules [20]. Cieslak et al. presented an interactive method for creating and calibrating developmental plant models for maize and canola based on the L-system [21]. With increasing research on plant growth systems, different plant growth models have been proposed. De Reffye et al. developed the AMAP plant growth software system using the reference axis technique, which is another popular method for virtual plant modeling [22]. The reference axis technique uses a finite automaton to simulate plant morphology, and plant growth and development processes were obtained by Markov chain theory and state transition diagrams. Mündermann et al. constructed successively quantitative models of plant topology and organs based on plant growth patterns and empirical data [23]. Jallas et al. established a coupled model that integrates plant architectural modeling and physiologically based modeling to virtualize the cotton growth process [24]. Dornbusch et al. used intercepted apical cones to simulate leaf sheaths and internodes of spring barley and ellipsoidal bodies to simulate spikelet seeds [25]. Espana et al. used a two-dimensional rectangular plane to approximate the leaf blade and mapped different parts of the leaf into a 3D space to achieve 2D and 3D morphological simulation of the maize leaf [26]. Based on the biomechanics of cell growth, Abera et al. developed tissue generation algorithms to study the influence of leaf anatomy on photosynthesis [27, 28]. Although virtual crop visualization has achieved tremendous improvement, the major limitation of the established plant models is that the plant models are not realistic in morphology, color, and texture, and cannot be used for precise crop visualization.

Generative adversarial networks (GAN) [29], proposed by Ian Goodfellow, have revolutionized the deep learning field. GAN is one of the most widely used techniques for image generation. It generates fake data consistent with its distribution by continuously approximating the data distribution of real samples. The network includes two parts: generator G and discriminator D. The goal of G is to generate fake samples by imitating the data distribution of real samples, and the goal of D is to make judgments on the authenticity of the samples. In the training process, G and D are constantly pitted against each other and the performance of G and D is continuously optimized. Consequently, the generator can generate highly realistic images. DCGAN (deep convolutional generative adversarial networks) [30] proposed by Alec Radford et al. is an extension of GAN, in which the authors combined GAN and CNN to improve the ability of the GAN to learn image representations. GAN has been widely used in various academic fields. In the crop research field, GAN applications are mainly focused on plant dataset augmentation. To alleviate the lack of training data in plant phenotyping, Giuffrida et al. used generative adversarial networks to expand the dataset and improved the CNN accuracy for detecting and segmenting Arabidopsis [31]. Espejo-Garcia et al. used GAN to create artificial tomato images and improved weed recognition accuracy [32]. Lu et al. enhanced the dataset by generating pest images using GAN to improve the accuracy of the CNN classifier model for pest classification [33]. Barth et al. used an unsupervised recurrent GAN for unpaired image-to-image translation to generate segmented bell pepper images from bell pepper images, reducing the manual labeling task [34]. Kerdegari et al. utilized a semisupervised GAN to generate multispectral images of agricultural fields, demonstrating the potential of GAN-based methods for multispectral image classification [35]. One advantage of GAN is that the generated virtual images are very realistic. Thus, GAN networks have the potential to be applied in crop visualization research to produce highly realistic virtual crops.

This paper aimed to generate virtual crops using phenotypic information as input. Specifically, the phenotypic traits for each crop were presented as a feature vector. In other words, a conditional image synthesis method that is conditioned on a feature vector, other than conditioned on a single label describing the overall image class or other images [36–38], was needed. Text-to-image generation is one of the classical tasks in computer vision, which aims to generate specific images by providing textual descriptions. StackGAN [39] proposed by Zhang et al. is a widely used text-to-image technique that can generate highly realistic images from text. StackGAN-v2 [40] is the improved version of StackGAN, which improved the training stability and the quality of the generated images. Specifically, in StackGAN-v2, text description information was first encoded and converted into vectors, and then the vectors were input into the generators to produce images. In this study, we introduced the idea of text-to-image conversion of StackGAN-v2 to transform crop phenotypic traits into crop images, with necessary modifications to the original model to make the model conditioned on a vector other than text. A GAN-based crop visualization tool named CropPainter was proposed. Given phenotypic traits, CropPaniter can generate realistic crop images. Specifically, we

1. Provided an image analysis pipeline for building the training dataset.
2. Developed a GAN-based crop visualization tool.
3. Evaluated the performance of the method for visualizing rice panicles (organ level).
4. Extended the method for visualizing crop plants (individual plant level), including rice, maize and cotton plants.

## Materials and methods

### Image acquisition

CropPainter was first developed for crop generation at the organ level. Rice panicles were chosen in this study as an example of virtual plant organ generation. To build the panicle dataset, 212 rice materials with 4 replications were used in this study. The rice materials came from Zhenshan, Minghui and their hybrids. The rice plants were cultivated in plastic pots at the crop phenotyping platform of Huazhong Agricultural University in 2014. At the filling stage, rice plant images were collected by a high-throughput imaging system [41]. For each rice plant, 15 side-view images of different angles were acquired.

To test the general applicability of CropPainter, CropPainter was extended for visualizing crop plants, including rice, maize and cotton. The rice plant images were acquired from the same rice materials as the panicle dataset, except that the rice plant dataset was captured at the seedling stage. To build the maize plant dataset, the maize plant images were acquired from 696 maize accessions with 4 replications at the seedling stage in 2019. The images of the cotton plant dataset were acquired from 200 cotton accessions with 3 replications at the seedling stage in 2017. All crop plants, including rice, maize and cotton, were cultivated in plastic pots at the crop phenotyping platform of Huazhong Agricultural University.

### Model training

CropPainter was trained on a computer with Ubuntu 16.04 and an NVIDIA GeForce RTX 2070s graphics card with 8 GB memory. The software environments were based on Python 2.7, which uses PyTorch, TensorBoard, Python-Dateutil, EasyDict, Pandas, and Torchfile. The Adam optimizer (lr = 0.0002, beta_1 = 0.5, beta_2 = 0.999, epsilon = 10e-8) was used in the training.

## Results

### The overall CropPainter flowchart

**Fig. 1:**
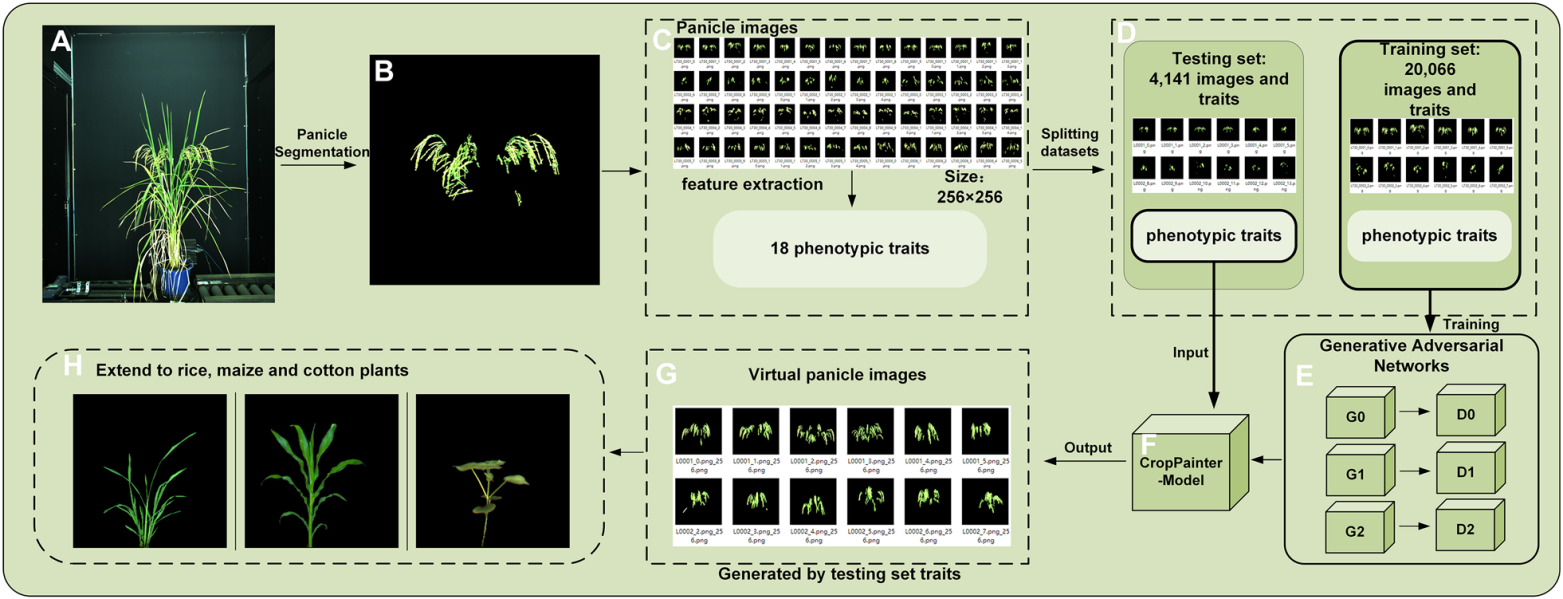
The overall flowchart. **A** Original RGB rice image. **B** Rice panicle image after segmentation. **C** Rice panicle image datasets along with phenotypic traits extracted by LabVIEW. **D** Randomly dividing the datasets into a training set and testing set. **E** Network Training. **F** Trained CropPainter model. **G** Generating virtual rice panicle images from traits in the testing set. **H** Extending CropPainter to visualize rice, maize and cotton plants

The overall CropPainter flowchart is shown in Fig. 1. The rice images (Fig. 1A) were first segmented to obtain panicle images (Fig. 1B). The panicle images were then analyzed to build the datasets for training and testing the CropPainter model (Fig. 1C, D). The training set, including 20,066 images and their corresponding phenotypic traits, was fed into the GAN model to train CropPainter (Fig. 1E, F). Phenotypic traits extracted from the 4,141 testing images were then input into the trained CropPainter. Virtual panicle images generated by the CropPainter were then evaluated to test the CropPainter performance (Fig. 1G). Finally, CropPainter was extended for the visualization of maize, rice and cotton plants (Fig. 1H).

### Image processing and phenotypic trait extraction

**Fig. 2:**
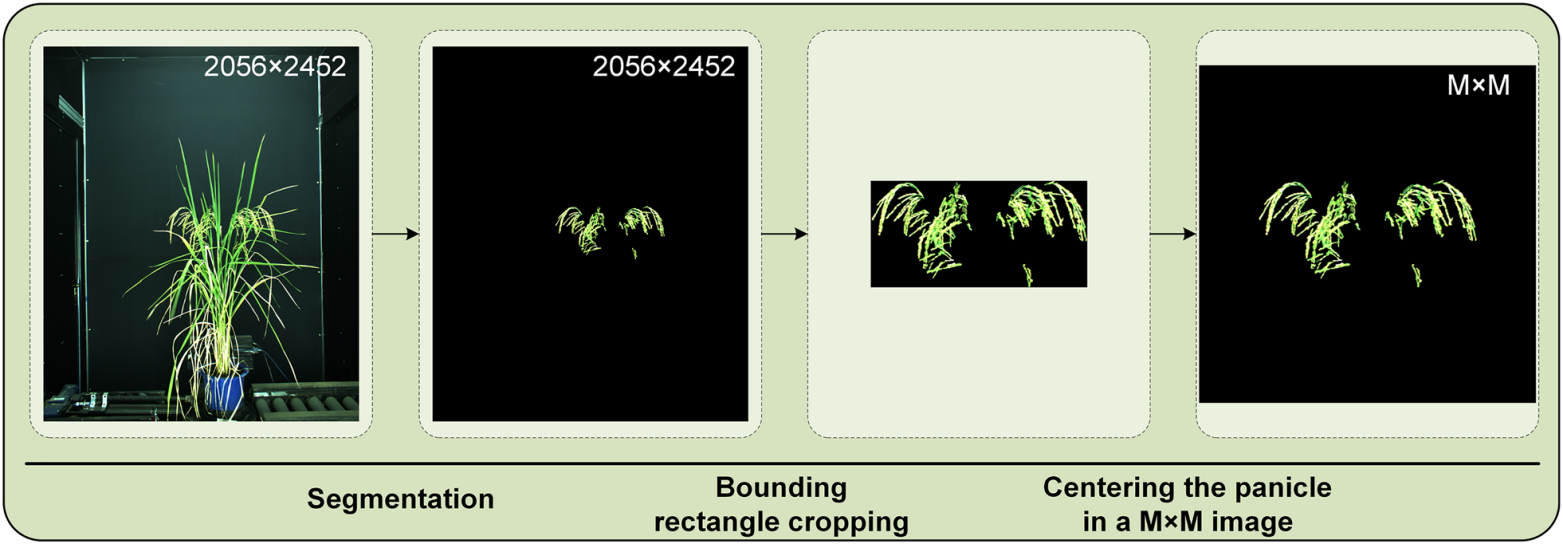
Image preprocessing flowchart

To obtain the rice panicle images for building the panicle dataset, the deep semantic segmentation model SegNet [42] was used to segment the panicles from the rice images. Subsequently, the panicle regions were cropped by detecting the minimum bounding rectangle of the panicle regions. The maximum height or width of the bounding rectangle among the datasets was then calculated and denoted as M. Then, the cropped image containing the panicle regions was centered in an image with a resolution of M×M (Fig. 2). Then, the images were resized to 256 × 256 to fit the GAN model. Finally, 24,207 images were prepared and divided randomly into two sets: a training set (20,066 images) and a testing set (4,141 images).

For the maize, rice and cotton plant datasets, the images went through a similar image processing flowchart. To fully express the maize plant characteristics, for each maize, only the image with the largest width among 20 side-view images was selected. For rice and cotton plants, all side-view images from different angles were used. In total, the numbers of images for the rice plant dataset, maize plant dataset and cotton plant dataset were 12,634, 11,064, and 7,280, respectively. The datasets were divided randomly into training sets and testing sets. The number of images and varieties in each dataset are listed in Table 1.

**Table 1:** The number of images and varieties used in each dataset

Datasets	Training sets	Testing sets	Varieties
Panicle	20,066	4,141	212
Rice plant	11,218	1,416	212
Maize plant	10,081	983	696
Cotton plant	6,680	600	200

After image processing, 18 phenotypic traits were extracted using LabVIEW 2015 (National Instruments, Austin, USA), including mean value (M), standard error (SE), smoothness (S), third moment (MU3), uniformity (U), entropy (E), total projected area (TPA), height of the bounding rectangle (H), width of the bounding rectangle (W), the ratio of total projected area and circumscribed box area (TBR), the circumscribed box area (CBA), the ratio of total projected area and hull area (THR), the ratio of perimeter and total projected area (PAR), fractal dimension with image cropping (FDIC), perimeter (P), green projected area (GPA), yellow projected area (YPA), and the ratio of total projected area and yellow projected area (YTR) [43].

### Crop visualization based on GAN

In our research, to visualize the phenotypic crop traits, we proposed a GAN-based method, namely, CropPainter, which takes the phenotypic crop traits as input and generated its corresponding crop image. StackGAN-v2 is a commonly used model for text-to-image generation that generates images from the input text. StackGAN-v2 first encodes the input text into multidimensional vectors and then feeds the encoded vector to the generator. The StackGAN-v2 comprises three discriminators and three generators in a tree structure to generate images of different scales.

**Fig. 3:**
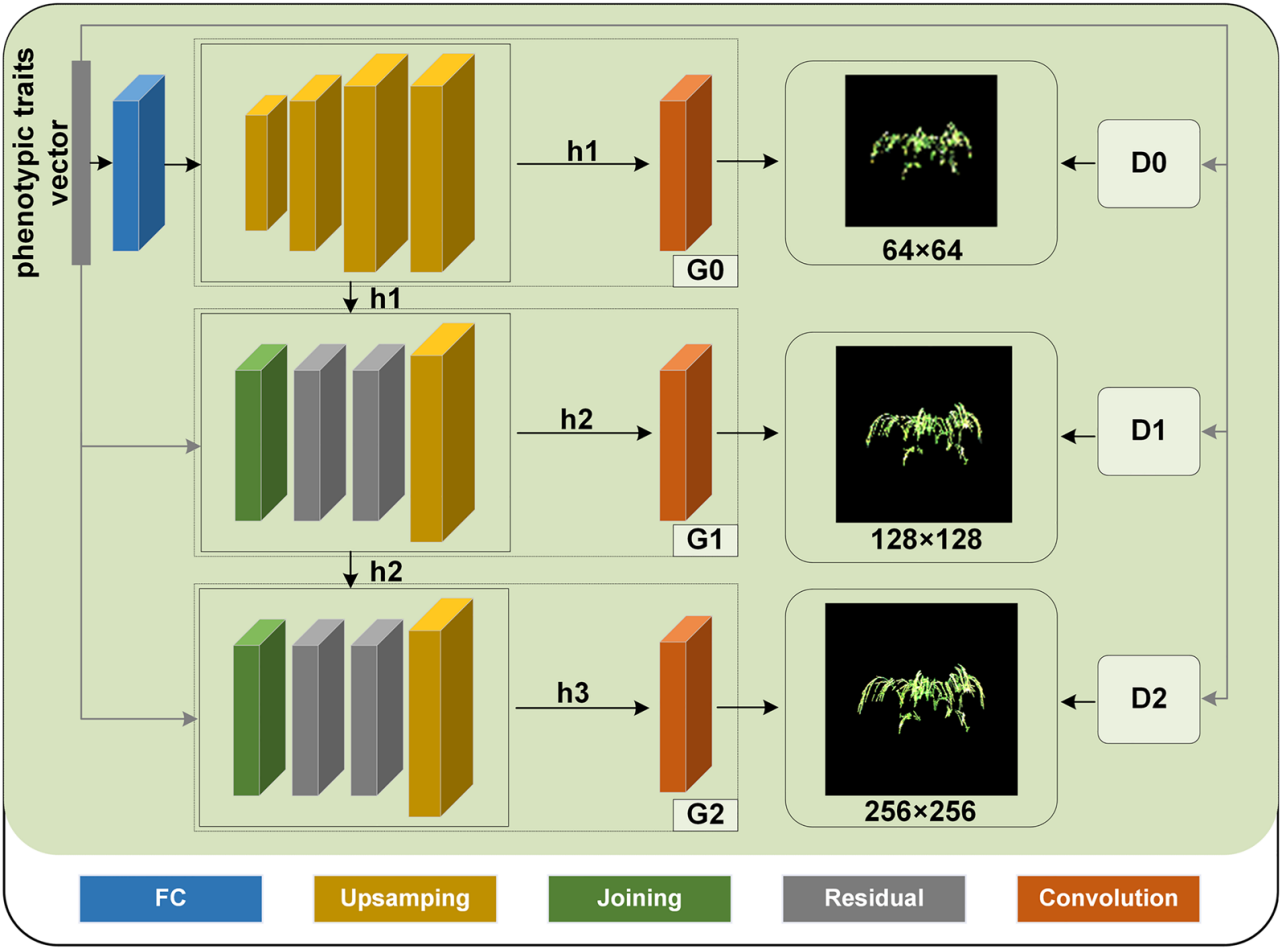
CropPainter network structure

In this study, we improved the StackGAN-v2 as the basic structure of CropPainter. Specifically, instead of the text embedding method, we used our own encoding approach to encode the phenotypic traits. The text embedding block in StackGAN-v2 was used to convert the text description into vectors. However, the phenotypic information in this study was already presented as vectors. Moreover, the scale of the phenotypic traits varied greatly in our datasets. Therefore, the input phenotypic traits were first normalized by a min-max normalizer, which linearly rescaled each trait to the [0,1] interval. The normalization was also beneficial for speeding up the model and improving the performance. For these reasons, we replaced the original text embedding block with normalization. In addition, the conditioning augmentation network (CA network) in StackGAN-v2 was removed in our study because the CA network adds unnecessary randomness, which is not beneficial for accurately generating crop images by phenotypic traits. The normalized phenotypic traits were then input into the generators as the condition vector. For the panicle dataset and cotton dataset, all 18 phenotypic traits were used as the condition vector. For the rice plant dataset and maize plant dataset, the YPA and YTR were removed, and the remaining 16 traits were used for image generation because there were almost no yellow components in rice plant images and maize plant images. The input of the next generator was formed by concatenating the hidden feature vector h by the previous generator and the phenotypic traits vector along the channel dimension. The discriminator was used to distinguish between real and fake images and determine whether the generated crop image matched the input phenotypic traits (Fig. 3). For the network structure, the generator in the first stage was composed of a fully connected block, four upsampling blocks, and a convolutional layer. The fully connected block contains a linear layer and a batchnorm layer. Each upsampling block contains an upsampling layer, a convolutional layer and a batchnorm layer. The hidden feature vector h1 output by the upsampling block was passed to the second-stage generator in subsequent training, which generated a low-resolution crop image with a dimension of 64 × 64 through the convolutional layer. The second-stage and third-stage generators were composed of a joint block, two residual blocks, an upsampling block, and a convolutional layer. The joint block included a convolutional layer and a batchnorm layer. Each residual block contained two convolutional layers and batchnorm layers. Phenotypic trait vector and h1 were concatenated along the channel dimension and passed through a convolutional layer, which outputs the hidden feature vector h2. After that, h2 was fed to the generator of the second stage, and a crop image with a dimension of 128 × 128 was generated. Finally, the h2 and phenotypic trait vectors were input into the third stage to generate a crop image with a dimension of 256 × 256, which was deemed the final virtual image generated by CropPainter.

### Evaluation of crop visualization

After training, the phenotypic traits of the testing sets were input into the trained model to generate the crop images. To evaluate the effectiveness of our method for crop visualization, we evaluated our model in two aspects: visual fidelity and prediction accuracy.

### Visual fidelity

The quality and fidelity of the virtual images generated by CropPainter were evaluated by calculating the structural similarity (SSIM) and the Frechet inception distance (FID) score [44, 45]. The SSIM value is a number between 0 and 1, indicating the similarity of the two images in terms of brightness, contrast and structure. A higher SSIM value indicates a higher similarity between the two images. The FID value calculates the distance between the real image and the virtual image in the feature space. The smaller the FID value is, the higher the quality of the virtual image. The computational formulas for SSIM and FID are provided in Eqs. 1 and 2.

1 $$SSIM(x,y)=\frac{(2{\mu }_{x}{\mu }_{y}+{c}_{1})({\sigma }_{xy}+{c}_{2})}{({\mu }_{x}^{2}+{\mu }_{y}^{2}+{c}_{1})({\sigma }_{x}^{2}+{\sigma }_{y}^{2}+{c}_{2})}$$

2 $${d}^{2}(F,G)={\left|{\mu }_{x}-{\mu }_{y}\right|}^{2}+tr\left[\sum x+\sum y-2{\left(\sum x\sum y\right)}^{1/2}\right]$$

where x and y are the real image and virtual image, respectively. μ_x_ and μ_y_ are the mean values of images x and y, respectively. σ_x_ and σ_y_ represent the standard deviation of the two images, respectively, and σ_xy_ is the covariance of x and y. ∑x and ∑y are the covariance matrices of the two images.

### Prediction accuracy

To evaluate the prediction accuracy of CropPainter, the phenotypic traits of the generated images (hereafter denoted as virtual traits) were calculated and compared with the phenotypic traits of the real images (hereafter denoted as real traits) in the testing set. The correlation coefficient and cosine similarity between virtual traits and real traits were computed to assess the accuracy of the model in terms of image phenotypic trait prediction. The correlation coefficient is the Pearson product moment correlation coefficient, which is a dimensionless index ranging from − 1 to 1 and reflects the degree of linear correlation between two datasets. Cosine similarity measures the magnitude of the difference between two samples by the cosine of the angle between the two vectors in the vector space. The computational formulas for the correlation coefficient and cosine similarity are provided in Eqs. 3 and 4.

3 $$r=\frac{\sum (x-\stackrel{-}{x})(y-\stackrel{-}{y})}{\sqrt{\sum {(x-\stackrel{-}{x})}^{2}\sum {(y-\stackrel{-}{y})}^{2}}}$$

4 $$\text{cos}\theta =\frac{{\sum }_{i=1}^{n}\left({x}_{i}{y}_{i}\right)}{\sqrt{{\sum }_{i=1}^{n}{\left({x}_{i}\right)}^{2}}\sqrt{{\sum }_{i=1}^{n}{\left({y}_{i}\right)}^{2}}}$$

where $x$ is the virtual traits, $y$ is the real traits, and $\stackrel{-}{x}$ and $\stackrel{-}{y}$ represent the mean values of the virtual traits and the real traits, respectively. n is the number of phenotypic traits used for trait-to-image crop visualization.

### Virtual panicle generation by CropPainter

**Fig. 4:**
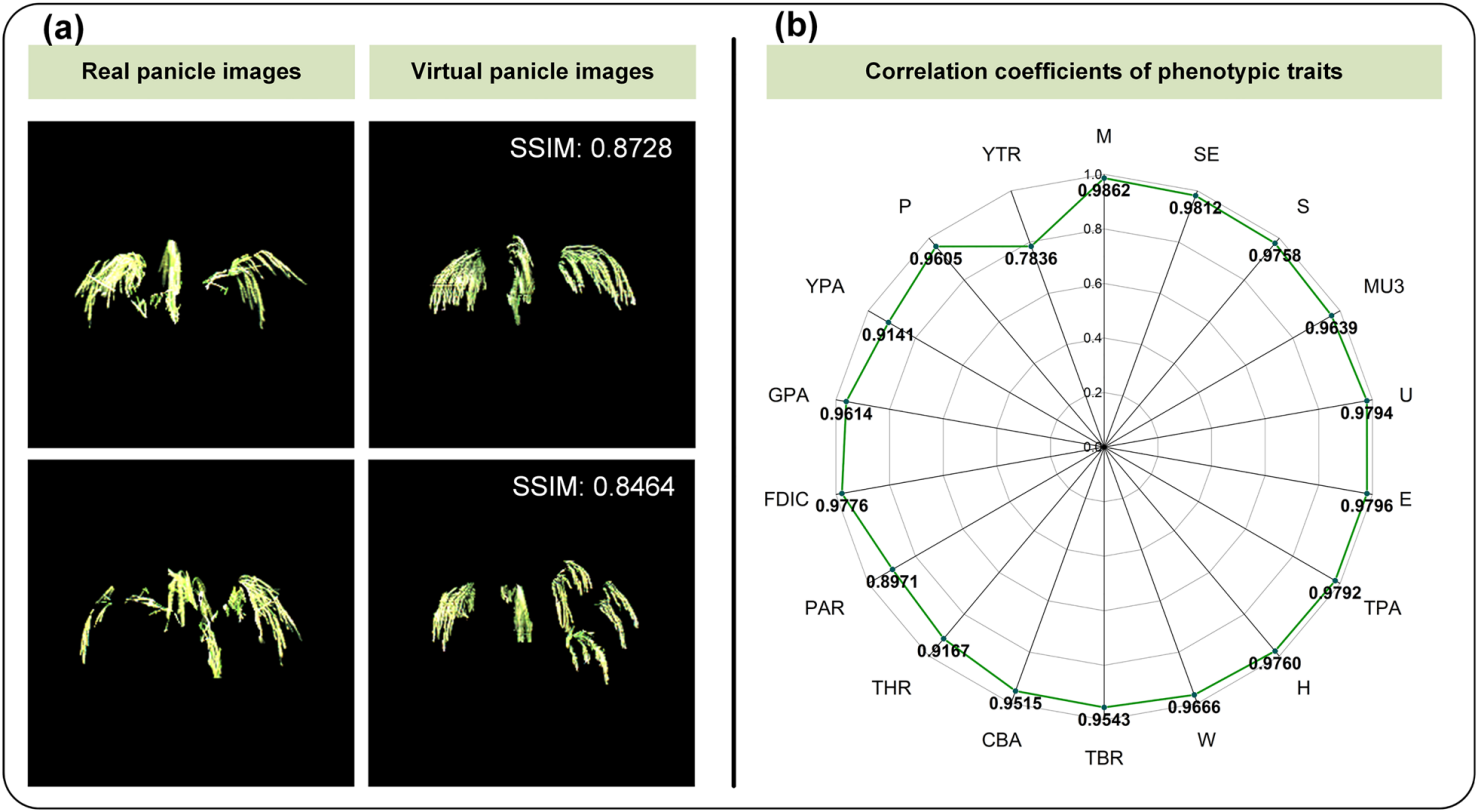
Panicle visualization results. **a** Generated virtual panicle images and the corresponding real panicle images. **b** Correlation coefficients of 18 phenotypic traits (real traits versus virtual traits)

Eighteen phenotypic traits extracted from 4,141 panicle images in the testing set were used as the CropPainter input, and the generated virtual panicle images were used to evaluate the CropPainter performance for panicle image generation. The average SSIM and FID values between the generated virtual images and real images reached 0.8322 and 22.82, respectively, which proved that the generated virtual images were realistic and similar to the corresponding real images. Figure 4a shows two examples of virtual panicle image generation.

In addition, the phenotypic traits of the generated virtual images (virtual traits) were extracted and compared with the phenotypic traits of the testing images (real traits). The mean value of correlation coefficients for the 18 traits was 0.9512, with the highest correlation coefficient reaching 0.9861 (Fig. 4b). The mean value and the standard deviation of cosine similarity of traits among the test dataset were 0.9881 and 0.0091, respectively.

### Virtual crop plant generation by CropPainter

**Fig. 5:**
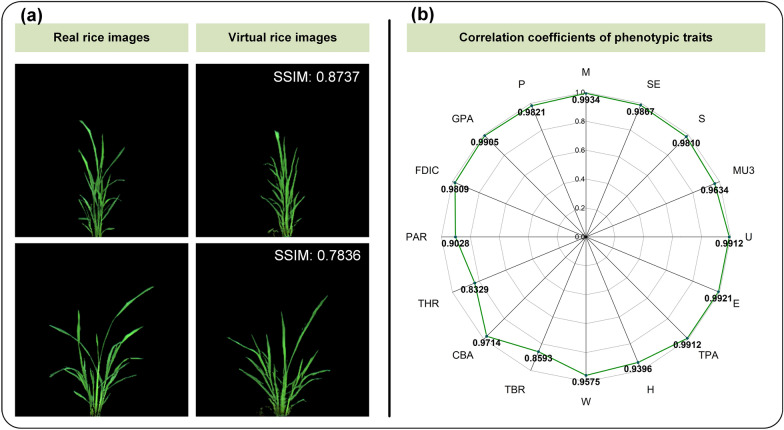
Rice plant visualization results. **a** Generated virtual rice images and the corresponding real rice images. **b** Correlation coefficients of 16 phenotypic traits (real traits versus virtual traits)

**Fig. 6:**
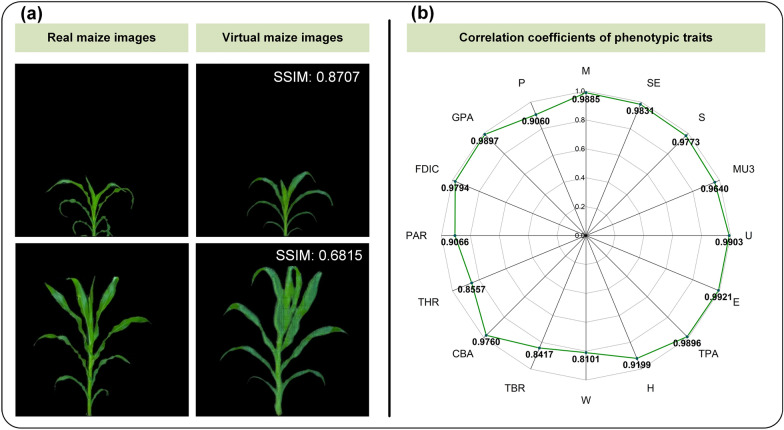
Maize plant visualization results. **a** Generated virtual maize images and the corresponding real maize images. **b** Correlation coefficients of 16 phenotypic traits (real traits versus virtual traits)

**Fig. 7:**
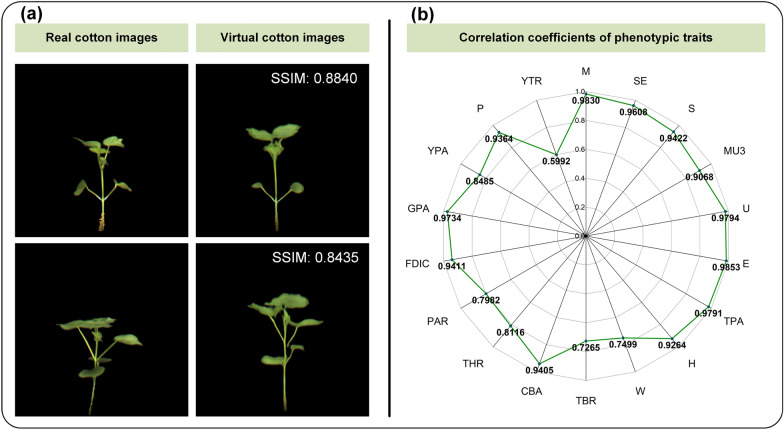
Cotton plant visualization results. **a** Generated virtual cotton images and the corresponding real cotton images. **b** Correlation coefficients of 18 phenotypic traits (real traits versus virtual traits)

To validate the generality of CropPainter, CropPainter was extended to generate virtual images of rice plants, maize plants and cotton plants. Figures 5a, 6a, 7a show examples of virtual image generation of rice plants, maize plants and cotton plants, respectively. Correlation analysis of virtual traits and real traits for rice plants, maize plants and cotton plants proved that the CropPainter achieved good performance in visualizing crop plants (Figs. 5b, 6b, 7b).

Table 2 illustrates the CropPainter performance in terms of visual fidelity and prediction accuracy. Generally, CropPainter had good applicability to visualizing different crops. Among the crop visualizations, CropPainter performed relatively worse on cotton plant visualizations. The main reason was that the number of images in the cotton plant dataset was approximately half of that in the rice plant dataset and maize plant dataset.

**Table 2:** Performance evaluation of CropPainter for visualization of panicle, rice plant, maize plant and cotton plant

Datasets	SSIM		FID	correlation coefficient		cosine similarity	
	Mean value	Standard deviation		Mean value	Standard deviation	Mean value	Standard deviation
Panicle	0.8322	0.0471	22.82	0.9512	0.0474	0.9881	0.0091
Rice plant	0.8583	0.0551	13.02	0.9572	0.0481	0.9942	0.0167
Maize plant	0.8550	0.0728	38.10	0.9418	0.0588	0.9938	0.0088
Cotton plant	0.8591	0.0370	61.02	0.8882	0.1065	0.9962	0.0063

### Software development

For the convenience of readers using our method, we created a UI interactive interface for CropPainter and packaged the project as executable software on the Windows 10 system. Readers can generate virtual crop images from trait information using this software. An additional video file shows the use of the software in more detail [see Additional file 1]. The software along with detailed instructions are available at http://plantphenomics.hzau.edu.cn/usercrop/Rice/download.

## Discussion

Traditional crop visualization methods cannot truly reflect crop characteristics. For instance, the existing methods usually use prior shapes to simulate plant organs. However, the exact organ shapes were different among different varieties and even among the same plant. For example, the shape of the leaves at different locations on the maize plant can be very different. GAN provides a new tool for digital crop visualization. Applying GAN for crop visualization using crop phenotypic information is a new idea. By improving the StackGAN-v2 structure, we introduced a novel method for trait-to-image crop visualization. The greatest advantage of our approach is that the developed crop visualization models have no constraints on the morphology, color and texture of the crop. Consequently, the generated virtual crops were highly realistic. In conclusion, the proposed CropPainter method is fundamentally different from traditional crop visualization methods.

To test the universality of our method, we carried out a series of experiments to verify it. We validated the CropPainter for crop visualization at both the organ level (rice panicles) and whole plant level (rice plant, maize plant and cotton plant), in which rice and maize were chosen as representatives of monocotyledons and cotton plants were selected as representatives of dicotyledons. Rice is a multitiller crop, and maize is a single-tiller crop. The results showed that our method had good adaptability to different crops. Moreover, the number and type of phenotypic traits used for virtual image generation can be adjusted conveniently, which indicates the flexibility of our method in the application of crop visualization. The significant contribution of our visualization method is that the generated virtual image is realistic and highly consistent with the input phenotypic traits. Consequently, CropPainter can also be used for precise image augmentation, which can create diverse and specified images for image classification tasks.

Accurate phenotypic trait extraction is essential for crop visualization using CropPainter. Inaccurate image segmentation leads to error in phenotypic trait calculation and thus result in an unsatisfying visualization. The number of images in the training dataset is another key factor for crop visualization. Generally, a larger sample size will produce a better visualization model. In our experiments, the accuracy of cotton plant image generation is relatively low. The main reason is that the number of images in the cotton dataset was relatively small, which was only a quarter of the number of images in the panicle dataset. In addition, unlike other traits, the color-related traits, including YPA and YTR, involved the segmentation of yellow and green components, which brought additional errors. Moreover, the color of the cotton stem close to the soil was yellow and very similar to that of the soil, which made it difficult to distinguish the cotton stem from the soil. Consequently, larger errors may have occurred in YPA and YTR extraction compared with other traits, which eventually resulted in lower correlation coefficients between the virtual color-related traits and the real color-related traits. A larger number of datasets and more precise image segmentation methods can improve the accuracy and similarity of CropPainter for virtual crop image generation. In addition, more samples are needed if the structure, shape or texture of the crop is complex or the dataset has a high diversity; for instance, the dataset has a large number of varieties.

Although the presented CropPainter performed well in crop visualization, it also had limitations. For example, CropPainter can only generate images with a resolution of 256 × 256 for this version. For this reason, only the visualization of crop plants at the seedling stage was tested in this study. Crop visualization tools that can generate virtual crop images with higher resolution will be developed in our future work, and the visualization of crop plants at later growth stages, for instance, the maturity stage, will be investigated. In addition, CropPainter can only generate a two-dimensional virtual crop. Three-dimensional virtual crop visualization tools will be studied in the future. This study generated virtual crop images based on crop phenotypic traits. However, the CropPainter input was not limited to phenotypic traits. Other crop omics datasets, such as genetic information, environmental information and combinations of different omics data, can also be used for generating virtual crops.

## Conclusion

This study proposed a novel trait-to-image crop visualization tool, namely, CropPainter, which can generate highly realistic and accurate virtual crop images based on crop phenotypic information. Our model also has the advantages of wide applicability and strong flexibility. To the best of our knowledge, there is no research available on crop phenotype visualization using GAN. Our method provides a completely novel idea for crop visualization and may serve as a tool for virtual plant research.

## Supplementary Information

**Additional file 1:** Instruction video for the CropPainter visualization software.

## Data Availability

Supplementary files for this article, which include datasets, trained models, software as well as the source codes used in this study, are available on website: http://plantphenomics.hzau.edu.cn/usercrop/Rice/download and https://github.com/zhwang-hzau/CropPainter-master.

## Abbreviations

GAN: Generative adversarial network
CNN: Convolutional neural network
G: Generator
D: Discriminator
